# Barriers and Facilitators that Influence on Adopting Healthy Lifestyles in People with Cardiovascular Disease

**DOI:** 10.17533/udea.iee.v39n3e04

**Published:** 2021-11-05

**Authors:** Jessica Natalia Saavedra Espinosa, Martha Yelitza Rodríguez Malagón, Sara Pamela Londoño Granados, Oscar Stiven Alméziga Clavijo, María Camila Garzón Herrera, Luz Patricia Díaz-Heredia

**Affiliations:** 1 Nurse. Faculty of Nursing, Universidad Nacional de Colombia. Bogotá, Colombia Email: jnsaavedrae@unal.edu.co Universidad Nacional de Colombia Universidad Nacional de Colombia Bogotá Colombia jnsaavedrae@unal.edu.co; 2 Nurse. Faculty of Nursing, Universidad Nacional de Colombia. Bogotá, Colombia Email: myrodriguezm@unal.edu.co Universidad Nacional de Colombia Universidad Nacional de Colombia Bogotá Colombia myrodriguezm@unal.edu.co; 3 Nurse, Specialist in Public Health Administration. Faculty of Nursing, Universidad Nacional de Colombia. Bogotá, Colombia Email: saplondonogr@unal.edu.co Universidad Nacional de Colombia Universidad Nacional de Colombia Bogotá Colombia saplondonogr@unal.edu.co; 4 Nurse, Master’s in clinical epidemiology (C). Faculty of Nursing, Universidad Nacional de Colombia. Bogotá, Colombia Email: osalmezigac@unal.edu.co Universidad Nacional de Colombia Universidad Nacional de Colombia Bogotá Colombia osalmezigac@unal.edu.co; 5 Nurse, Master’s in clinical epidemiology (C). Faculty of Nursing, Universidad Nacional de Colombia. Bogotá, Colombia Email: mcgarzonh@unal.edu.co. Corresponding author Universidad Nacional de Colombia Universidad Nacional de Colombia Bogotá Colombia mcgarzonh@unal.edu.co; 6 Nurse, PhD in Nursing. Faculty of Nursing, Universidad Nacional de Colombia. Bogotá, Colombia Email: lpdiazh@unal.edu.co Universidad Nacional de Colombia Universidad Nacional de Colombia Bogotá Colombia lpdiazh@unal.edu.co

**Keywords:** healthy lifestyle, nursing, health promotion, cardiovascular diseases., estilo de vida saludable, enfermería, promoción de la salud, enfermedades cardiovasculares., estilo de vida saudável, enfermagem, promoção da saúde, doenças cardiovasculares.

## Abstract

**Objective:**

To measure lifestyle changes and describe the barriers and facilitators perceived that influence on adopting healthy lifestyles in people with cardiovascular diseases.

**Methods:**

Mixed study of concurrent execution in the public health center of the municipality of Tausa, Colombia. The quantitative phase corresponded to a longitudinal analytical method in which the FANTASTICO instrument was applied to 28 patients in this program between 0 and 120 days after a brief nursing intervention (face-to-face meetings and telephone calls). The qualitative phase was carried out with a micro-ethnographic approach applying a semi-structured interview to 12 out of 28 participants, 120 days after the intervention.

**Results:**

During the quantitative phase, a statistically significant change (*p* < 0.05) was the improvement of the total score and in the domains of activity, type of personality and insight between day 0 and 120. During the qualitative phase, 13 categories arose regarding barriers and facilitators to adopt a healthy lifestyle: four facilitators and one barrier for physical activity, three facilitators and three barriers for feeding, and two facilitators for stress management. By integrating the results, it is possible to explain that, for the change in eating behaviors, physical activity and stress management, personal biological and psychological factors, interpersonal and situational influences coincide with the assumptions and propositions of the Health Promotion Model by Nola Pender.

**Conclusion:**

The participants’ lifestyles changed positively in three of the domains and the total of the instrument, which can be explained by simultaneous triangulation, by the facilitators and perceived barriers as influential on adopting behaviors to acquire a healthy lifestyle.

## Resumen

**Objetivo:**

Medir el cambio en el estilo de vida y describir las barreras y los facilitadores percibidos que influyen en la adopción de un estilo de vida saludable en personas con enfermedad cardiovascular.

**Métodos:**

Estudio mixto de ejecución concurrente en el centro de salud público del municipio de Tausa, Colombia. La fase cuantitativa correspondió a un método analítico longitudinal, en el que se aplicó el instrumento FANTÁSTICO a 28 pacientes del programa a los 0 y 120 días de realizada una intervención breve de enfermería (encuentros presenciales y llamadas telefónicas). La fase cualitativa se realizó con un abordaje micro-etnográfico aplicando una entrevista semiestructurada a 12 de los 28 participantes, después de 120 días de realizada la intervención.

**Resultados:**

En la fase cuantitativa, se encontró un cambio estadísticamente significativo (*p*<0.05) en la mejoría del puntaje total y en los dominios de actividad física, tipo de personalidad e introspección entre el día 0 y 120. En la fase cualitativa, surgieron 13 categorías relacionadas con barreras y facilitadores para la adopción de un estilo de vida saludable: cuatro facilitadores y una barrera para la actividad física, tres facilitadores y tres barreras para la alimentación y dos facilitadores para el manejo del estrés. Al integrar los resultados, es posible explicar que, para el cambio en las conductas de alimentación, actividad física y manejo del estrés, inciden factores personales biológicos y psicológicos e influencias interpersonales y situacionales que coinciden con los supuestos y proposiciones del Modelo de Promoción de la Salud de Nola Pender.

**Conclusión:**

El estilo de vida de los participantes cambió positivamente en tres de los dominios y el total del instrumento, lo que puede ser explicado mediante la triangulación simultánea, por los facilitadores y barreras percibidos como influyentes en la adopción de conductas para la adquisición de un estilo de vida saludable.

## Resumo

**Objetivo:**

Medir a mudança no estilo de vida e descreva as barreiras e facilitadores percebidos que influenciam a adoção de um estilo de vida saudável em pessoas com doenças cardiovasculares.

**Métodos:**

Estudo misto de execução concorrente no centro de saúde pública do município de Tausa, Colômbia. A fase quantitativa correspondeu a um método analítico longitudinal, em que o instrumento FANTÁSTICO foi aplicado a 28 pacientes do programa aos 0 e 120 dias após uma breve intervenção de enfermagem (encontros presenciais e ligações telefônicas). A fase qualitativa foi realizada com abordagem microetnográfica. Uma entrevista semiestruturada foi realizada com 12 dos 28 participantes, 120 dias após a intervenção.

**Resultados:**

Na fase quantitativa, foi encontrada mudança estatisticamente significativa (*p* <0.05) na melhora da pontuação total e nos domínios atividade física, tipo de personalidade e introspecção entre os dias 0 e 120. Na fase qualitativa, surgiram 13 categorias relacionadas às barreiras e facilitadores para a adoção de um estilo de vida saudável: quatro facilitadores e uma barreira para atividade física, três facilitadores e três barreiras para alimentação e dois facilitadores para controle do estresse. Ao integrar os resultados, é possível explicar que, para a mudança nos comportamentos alimentares, atividade física e gerenciamento do estresse, afetam fatores biológicos e psicológicos pessoais e influências interpessoais e situacionais que coincidem com os pressupostos e proposições do Modelo de Promoção da Saúde de Nola Pender.

**Conclusão:**

A pontuação do FANTÁSTICO dos participantes mudou positivamente em três dos domínios e no total do instrumento, o que pode ser explicado pela triangulação simultânea, pelos facilitadores e pelas barreiras percebidas como influenciadoras na adoção de comportamentos para a aquisição de um estilo de vida saudável.

## Introduction

The World Health Organization (WHO) defines healthy lifestyle as a “way of life based on identifiable behavioral patterns, determined by the interaction between personal individual characteristics, social interactions, and socioeconomic and environmental life conditions”.(1) Different conducts have been studied that intervene in the conformation of a healthy lifestyle, among them, it highlights: having balanced nutrition, engaging regularly in physical activity, avoiding excess alcohol and eliminating cigarette smoking, which has been associated with higher life expectancy.(2) This theme represents great interest because adherence to healthy life habits impacts positively on the health and quality of life of people.(3)

The WHO states that an “optimal lifestyle to which all people can subscribe does not exist. Culture, income, family structure, age, physical capacity, home and work environment will make certain forms and conditions of life more attractive, feasible and suitable”.(1) This is why the prevalence of diseases and their repercussions are attributed not only to individual factors, but to interpersonal relations and life conditions, a perspective under which it considered that lifestyles and habits “are shaped by personal decision and by influence from our environment and social group”,(4) which is why adopting healthy practices also contemplates individual risk and vulnerability framed within society as factors that hinder or facilitate generation of changes in conduct toward healthy lifestyles.

In recent years, there is evidence of the growing expansion of chronic noncommunicable diseases (CNCD), which in 2019 caused 74% of the global deaths, being the principal cause of morbidity and mortality,(5) especially cardiovascular diseases, with 32.2% of deaths due to CNCD, having the highest proportion of deaths and disease burden in developing countries.(6) These diseases provoke alterations in the different spheres of humans, which impact on their quality of life and have a negative impact on them, their families, and society.

Cardiovascular diseases are the consequence of genetic, physiological, environmental and behavioral factors, with the last being closely related with their prevalence.(7) Thus, different interventions have been conducted to mitigate them, among them, the brief intervention, which is based on a counseling approach on harmful habits through a motivational interview, widely used to reduce excessive alcohol consumption, but with low documentation in the literature of its implementation in other behaviors that shape the lifestyle, whose coping strategies of coping allow greater effectiveness than traditional educational interventions.(8) Due to this, a quasi-experimental macro-project carried out in the municipality of Tausa, Cundinamarca, implemented this intervention between 2017 and 2018 to improve the lifestyles of people with cardiovascular disease, from which this study is derived, given that it is necessary to determine if a change in conduct took place in the individual and investigate the factors that make individuals carry out or not said change, which is why a mixed research was conducted with qualitative approach.

This is due to the predominance of research with a quantitative approach and the complicated approach to the social, economic and environmental conditions and influences that affect people's behavior, which results especially important to consider the perceptions of people regarding these aspects as barriers or facilitators to change, given that evidence in terms of qualitative studies is insufficient and quantitative studies do not allow profound understanding of its influence. Likewise, little has been found on the matter of mixed studies that investigate the effect, as well as the experience of people with regard to presumably effective interventions on the change in the behaviors that constitute the lifestyle, as is the case of the brief intervention by nursing.

Hence, it is of great interest for nursing to inquire on how personal factors, interpersonal relations, and life conditions influence on adopting a healthy lifestyle to boost actions that promote wellbeing in the individuals, their families, and the community, fostering acquisition of health-promoting conducts, as proposed by the Health Promotion Model (HPM) by Nola Pender, where nursing professionals constitute the principal agent in charge of motivating people to maintain or reach an optimal state of health.(9)

Thus, it is fundamental to approach the issue from a mixed focus that keeps in mind not only the lifestyle change, but the perceptions of people on the factors that facilitate or make difficult adopting conducts that impact positively on health. Due to this, this study was conducted, seeking to measure lifestyle change and describe the barriers and facilitators perceived that influence on adopting a healthy lifestyle in people with cardiovascular disease who received a brief nursing intervention.

## Methods

The present study has a mixed study of concurrent execution, which emerged from the research macro-project “Brief intervention in people with cardiovascular disease who have unhealthy behaviors and risky intake of alcohol”. The quantitative phase was approached with a longitudinal analytical method, which measured lifestyle change and the qualitative phase was carried out through a micro-ethnographic approach to identify the barriers and facilitators that influence on the adoption of a healthy lifestyle; subsequently, complementation between methods was carried out, under the mechanism of simultaneous methodological triangulation.

Data collection took place between October 2017 and April 2018, with 30 people enrolled in the chronic disease program from the Tausa Health Center participated in the pilot test of the brief intervention, implemented by the macro-project, of which two did not comply with some of the inclusion criteria: being > 18 years of age, people diagnosed with cardiovascular disease, have completed 120 days after the brief intervention, having adequate cognitive and verbalization capacity, and having the signed informed consent to participate in the study. Hence, the study included 28 people with cardiovascular disease who were part of the program.

It must be indicated that the brief intervention of the macro study was developed as a therapeutic recourse, seeking to promote behavioral changes through face-to-face meetings and phone calls that lasted from 5 to 20 min.(10) The individual was motivated to commit to the change in any modifiable risk factor, like - for example - alcohol intake, cigarette smoking, or lack of physical activity. The brief intervention was developed based on the phases of feedback, responsibility, advice, options menu and empathy, during the meeting on the first day and after 30 days; thereafter, follow up was conducted at 90 and 120 days to recognize the lifestyle change, time the literature shows as appropriate to recognize maintenance of new conducts. (11)

The quantitative phase applied the FANTASTICO questionnaire immediately before carrying out the brief intervention (pre-intervention) and 120 days after (post-intervention). This instrument was designed by the Department of Family Medicine at McMaster University in Canada, whose total score is categorized in five levels, which permit stratifying the lifestyle into excellent (85 to 100 points), good (70 to 84 points), regular (60 to 69 points), poor (40 to 59 points), and at risk (≤39 points). A score of “excellent” indicates that the lifestyle generates an optimal influence for health; “good” means it exerts an adequate influence for health; “regular” indicates that benefits and risks exist; “poor and danger exists” warns that the individual’s lifestyle implies greater risk for health.(12)

The questionnaire consists of 25 closed items that explore 10 domains on the physical, emotional, and social factors of lifestyle, which are: Family and friends (2 items), Activity (2 items), Nutrition (3 items), Tobacco (2 items), Alcohol (3 items), Sleep and stress (3 items), Type of personality (2 items), Insight (3 items), Conduction and work (2 items), and Other drugs (3 items). This questionnaire proposes three response options with a numerical value from 0 to 2 for each item of the domain, scored through a Likert-type scale, with a minimum score of 0 and maximum of 6 for the domains and a total score from 0 to 100. The instrument has been translated and validated in Colombia, showing adequate reliability.(12)

The statistical analysis used the *R* software and the *exactRankTests* library developed by Hothorn *et al*.,(13) applying the Wilcoxon Signed Rank non-parametric test to measure the lifestyle change over time in the total score of the instrument and per domain, using 95% confidence interval.

The qualitative phase conducted a semi-structured survey, applied after carrying out the brief intervention. The surveys were developed face-to-face and audio recorded for their subsequent transcription and analysis. Their transcription was done as they were collected, until reaching theoretical saturation, resulting in a total of 12 participants. The survey questions were evaluated by using the Flesch-Szigriszt Readability Index for health texts, through the INFLESZ software, (14) finding a rather easy degree of legibility on the INFLESZ Scale, which favored application of the survey, bearing in mind the low level of schooling of the participants.

This survey was composed of 13 questions that guided participants in identifying the goals agreed upon with respect to their health during the entire project and those they managed to comply or not, besides the general aspects that helped or hindered their fulfillment. In addition, it was aimed at identifying influence by the family, work, housing, environment, and health center on the change process to adopt a healthy lifestyle in the participants. After this, a qualitative analysis of content was performed to generate categories.

Finally, triangulation of the results was performed to describe how the facilitators and barriers perceived by the participants intervene in the change process to adopt a healthy lifestyle. For this, qualitative and quantitative results were analyzed separately, comparing them to identify their association, taking as referent the HPM by Nola Pender, with which the study seeks to comprehend why health promoting conducts are or are not adopted, considering both personal factors and interpersonal and situational influences that emerged from the qualitative analysis, associated with change in the total score and in the domains of the FANTASTICO questionnaire.

This study was approved by the University’s Research Ethics Committee and was authorized by the E.S.E. Centro de Salud de Tausa, Cundinamarca. During the investigation, the dignity, integrity and rights of the participants were safeguarded, in accordance with national and international regulations and the ethical principles established in Legislation 911 of 2004,(15) which dictates the provisions regarding deontological responsibility for the exercise of the nursing profession in Colombia. In compliance of article 6, had an informed consent signed by the participants, protecting their identity and using the information for merely academic purposes.

## Results

The sample was comprised of 21 women (75%) and seven men (25%), with mean age of 72 years, (minimum 50 years and maximum 93 years; SD = 10.49). With respect to place of birth, 75% were born in the municipality of Tausa and the majority lives in the rural zone (64.3%), this being the same percentage corresponding to socioeconomic level 1. As per level of schooling, the most frequent was incomplete primary (53.5%), followed by no educational level (25%). With relation to occupation, 75% were dedicated to the home; in terms of marital status, most were married (46.4%) or widowed (35.7%). All the individuals had arterial hypertension and were affiliated to the subsidized health scheme.

Regarding the quantitative phase, Table 1 presents the descriptive statistics and p value corresponding to the scores of each of the domains and of the total of the instrument. It may be noted that only the domains of Physical activity, Type of personality and Insight had a positive change with a statistically significant difference between the pre- and post-intervention (Wilcoxon Signed Rank; *p < 0.05*). For the total pre- and post-intervention scores of the FANTASTICO questionnaire, it was found that the participants changed to a healthy lifestyle, with a statistically significant difference (*p < 0.05*).

**Table 1:** Descriptive statistics and p value of the scores per domain and total of the FANTASTICO questionnaire according to moment of evaluation

Variables	Day	Minimum	Maximum	Mean	Median	Standard deviation	*p value*
Family and friends	0	1	4	3.429	4	0.920	0.6133
Family and friends	120	1	4	3.536	4	0.922	0.6133
Activity	0	0	4	2.25	3	1.798	0.0148
Activity	120	0	4	3.071	3	1.214	0.0148
Nutrition	0	2	6	4.464	5	1.036	0.1643
Nutrition	120	2	6	4.821	5	1.219	0.1643
Tobacco	0	3	4	3.964	4	0.189	1
Tobacco	120	2	4	3.929	4	0.377	1
Alcohol	0	4	6	5.786	6	0.499	0.75
Alcohol	120	3	6	5.821	6	0.612	0.75
Sleep and stress	0	2	6	4.571	5	1.259	0.1448
Sleep and stress	120	1	6	4.929	5	1.412	0.1448
Type of personality	0	1	4	2.536	2.5	1.036	0.0041
Type of personality	120	1	4	3.214	3.5	0.917	0.0041
Insight	0	1	6	3.964	4	1.374	0.0079
Insight	120	1	6	4.893	5.5	1.423	0.0079
Conduction and work	0	1	4	3.5	4	0.839	0.1289
Conduction and work	120	2	4	3.75	4	0.518	0.1289
Other drugs	0	3	6	5.571	6	0.790	0.0937
Other drugs	120	4	6	5.893	6	0.315	0.0937
Total score	0	48	98	81.21	78	10.712	0.0005
Total score	120	58	100	87.71	90	11.491	0.0005

It was found that prior to the brief intervention, according to the score of the FANTASTICO questionnaire, 50% reported a good lifestyle, followed by 39.3% with excellent, 7.1% as regular, and 3.6% as poor. With respect to the post-intervention, 75% reported excellent lifestyle, followed by 17.9% as good, 3.6% as regular, and 3.6% as poor, indicating a statistically significant positive change in the proportion of people with an excellent lifestyle between before and after the intervention (*p < 0.05*).

For the qualitative phase, upon applying the survey, the information was classified by following the six steps of the content analysis technique, thus, emerging 13 categories taken from the expressions and words referred by the participants and which were distributed between barriers and facilitators perceived. Taking as reference the conducts that conform a lifestyle, these were classified within the areas of feeding, physical activity, and stress management.

The following describe the barriers and facilitators perceived by the participants to adopt the conducts that comprise a healthy lifestyle.

### Facilitators for physical activity

***My family moves me*:** defined as the support provided by the family through companionship, motivation, and recommendations on doing exercise: *He invites me to go bring firewood or walk around for a while. (E3)*

***My activities keep me in shape*:** defined as the characteristic activities of the occupation that imply constantly engaging in physical activity: *Because of them one exercises and moves, one is concerned with seeing them, with milking them and everything and if not, one would have to sit around the house. (E10)*

***Here I can exercise*:** defined as the set of conditions of the physical space that surround the person and permit adopting continuous physical activity: *Given that I have the backyard, there I also do exercise, or here there is also a lot of open space, you just have to careful. (E11)*

*If I don’t stop, I stay fine*: defined as the aspects related with motivation, taste, purpose, or effort: *Have the purpose of walking and cycling for 30 minutes or more. (E4)*

### Barrier for physical activity

*My body limits me:* defined as the discomfort, pain, or physical limitation the individual manifests: *it is that my foot hurts, it’s like inside, like an internal bunion as it is called, but it was not operated because it was not on the sole of the foot, so it was not operated. (E4)*

### Facilitators for feeding

***Eating well with what we have and what we have we share*:** defined as their own financial means or an economic or material contribution provided by the family: *They help me, but to buy me the fruits and all that because they are working. (E11)*

***In my family we take care of each other and we collaborate with the food*:** defined as the support offered by the family, providing advice, care, or companionship and participating mutually: *My daughters also use to eat too much salt and we taught them that... and they also do the same, we all comply with it. (E7)*

***I eat better when I put my mind to it*:** defined as aspects related with the reasons, impulses, purposes, motivations, or tastes: *To diminish fat, that takes effort, but has to be done, yes, persuade yourself from those things to be able to overcome and see if you can achieve it. (E10)*

### Barriers for feeding

***Eating what you can with the money you have*:** defined as shortness or lack of a financial means that makes it difficult to adopt healthy eating: *Yes, if you buy the medicine, you don't have enough for food, and if you buy the food, you won't have any left for the medicine, it's not easy. (E6)*

***Eating poorly at work*:** defined as the circumstances derived from the wage-earning work activity, which make it difficult to adopt healthy eating: *Sometimes when helping customers one stops, for example, having lunch on time, or they are having lunch and I am in the store so they have lunch first and I attend here and then have lunch. (E5)*

*I think about changing what I eat, but there is something that defeats me:* defined as the negative perception of the state of health that prevents the use of the land to grow food, the presence of unfavorable emotions towards some healthy foods, or the need to eat more than is due: *Eating, since one feels hungry, then with always complications that tempts one into eating. (E4)*

### Facilitators for stress management

*When talking to my neighbor, every day is calmer:* defined as the companionship provided by a neighbor or friend, allowing individuals to diminish levels of tension in their lives: *Venting with someone whom you trust and, yes, that helped me a lot. (E9)*

***I get distracted and stay busy doing what I do*:** defined as the occupation of the person's time in activities at home, which facilitates stress management: *tasks around the house, household work. I have chickens, I have a calf and I take care of them […] they actually help me because they distract me. (E9)*

Upon completing the qualitative analysis process, it was identified that people refer to family support, company of neighbors, occupation activities, and taste or purpose as facilitators for conducts of regular physical activity, balanced feeding, and stress management; however, they perceived that lack or scarcity of money, occupation, appetite, physical limitations, among others are barriers in the change process.

## Discussion

This study, which sought to measure lifestyle change and describe the barriers and facilitators perceived that influence on adopting a healthy lifestyle, observed the lifestyle of the participants improved between the pre- and post-intervention. This may be explained from the facilitators perceived, like family support, company of neighbors, occupation activities, and taste or purpose, which helped to adopt regular physical activity regular, balanced feeding, and stress management. However, not all the participants reached an excellent lifestyle, given that they perceived as barriers the lack of money, occupation, appetite, physical limitations, among others.

It was found that physical activity increased the score in this domain, which is possible to associate with the facilitators mentioned by the participants, like the taste for physical activity, daily tasks, their personal effort, and family support, which seen from the HPM, can be interpreted as interpersonal influence due to the support provided by the family, situational influence due to the available options perceived in the occupation and the environment, and personal psychological factors due to the personal competence. As mentioned by Silva *et al*.,(16) facilitators exist that permit adopting physical activity, among them, factors from the interpersonal level that include social support networks; factors from the physical environment; and factors from the intrapersonal level, which correspond to the individual preferences and the combination with pleasant and useful activities, like daily activities. In spite of the change in this domain, not all the participants achieved the maximum score because the discomfort, ailments, and physical limitations evidenced in the perceived barrier made it difficult to perform regular physical activity, which is related with the biological personal factors in the HPM. This agrees with Silva *et al.,*(16) and Cortés(17) who indicate as barriers to engage in physical activity the current state of health and the very physiological aging process.

For nutrition, no significant change was noted, which is associated with barriers related with situational influence, given reportedly poor control in feeding due to their working conditions and lack or scarcity of money to acquire adequate foods for their health; and personal psychological factors, given that they mentioned dislike towards some healthy foods, temptation to eat, appetite and taste for unhealthy foods. However, there was an increase in the frequency of the maximum score, which may be associated with facilitators, like personal purpose and family support, corresponding in the HPM to personal psychological factors and las interpersonal influence, respectively.

A systematic review by Kelly *et al*.,(18) gathers different studies on healthy conducts and, specifically on feeding, establishes family support as facilitator, which is determinant to accept and maintain healthy feeding. Furthermore, it identifies sociocultural factors as barriers, including conditions at work. Likewise, the study establishes that low family wages implies lower consumption of fruits, vegetables and fiber, and mentions that psychological factors, like lack of capacity and motivation, as well as the ingrained preferences for unhealthy foods, hinder adopting the conduct.

Finally, stress management kept in mind change in the domains of: Sleep and stress, Type of personality, Insight, Family and friends, and Conduction and work for the integration with the results from the qualitative phase, which were only facilitators. The Sleep and stress domain evidenced that by day 120 frequency increased in the maximum score; the Family and friends domain showed that the frequency in the maximum score remained high both on day 0 as in day 120, which may be associated with the facilitator perceived for this conduct - described in the HPM, as the interpersonal influence of neighbors and friends, which highlights dialogue, talking and venting with others.

Likewise, for the Conduction and work domain, the frequency of the maximum score for days 0 and 120 remained high, which is associated with person’s time spent in distracting activities in the home, which corresponds in the HPM to a positive situational influence for stress management. Additionally, the scores obtained in the domains of Type of personality and Insight had a statistically significant change, which behave - according to the HPM - as personal psychological factors that can influence upon stress management, however, were not perceived by the participants as facilitators for this conduct.

It is scarce evidence regarding the factors that influence upon this behavior. A study by Barrier (19) about the participation of adults in physical-recreational activities, points to an association between physical activity and sociability, given that it implies a greater connection with their environment and, hence, greater social interaction, which facilitates stress management. Moreover, it exposes that older adults who engage in regular physical activity improve their self-esteem and social situation, interpreted as improvement in the physical, social, and psychological dimension. Nevertheless, it does not describe explicitly the influence exerted by interpersonal relations and activities of the occupation on stress management.

This research managed to identify the barriers and facilitators that influence on adopting healthy lifestyles in a rural population. The total score of the FANTASTICO questionnaire increased by day 120 after conducting the brief intervention, obtaining improved lifestyle, specifically, in the domains of Activity, Type of personality and Insight. Through triangulation, it was possible to associate and explain lifestyle change with the facilitators and barriers perceived, according with the Health Promotion Model by Nola Pender, establishing that change toward a healthy lifestyle is explained with the facilitators perceived by the participants; however, the maximum scores were not achieved in some domains, which may be associated with the barriers perceived in the change process.

The mixed focus permitted knowing the influence of personal characteristics, as well as the interpersonal relations and life conditions on adopting a healthy lifestyle, given that not only was change determined, but that the perception of the participants was considered as to factors that behave as barriers or facilitators for said change to take or not take place. Through this study, it was possible to know how the context influenced on individuals to achieve lifestyle modifications, to then propose future interventions that respond to the needs of the rural population to promote adoption of healthy conducts that improve their behaviors, supporting health care and enriching the nursing profession.

The evidence demonstrates that the interventions seeking to improve lifestyles focus generally on healthy people or with risk factors, which is not generalizable to individuals with cardiovascular diseases, which is why this study serves as base to carry out tertiary prevention strategies that permit behavioral change toward healthy conducts.

This research had limitations, such as sample size of the quantitative component, given that it does not represent a significant percentage of the study population; it must be highlighted that the results are applicable to individuals in chronic disease programs in the rural area because they were our study population.

Regarding the qualitative phase, some of the limitations were the possibility of ignoring or minimizing data, which was controlled with an independent work and thereafter a joint analysis by the researchers, to obtain greater interpretative and analytic wealth. Additionally, the study considered the possibility of a Hawthorne effect, given that the individual was aware of the intervention received, while the researcher’s effect was controlled, given that the initial measurement and the intervention were carried out as of the macro-project.
